# Concentration dependence of the sol-gel phase behavior of agarose-water system observed by the optical bubble pressure tensiometry

**DOI:** 10.1038/s41598-020-58905-8

**Published:** 2020-02-14

**Authors:** Nobuyuki Ichinose, Hodaka Ura

**Affiliations:** Department of Chemistry and Materials Technology, Faculty of Engineering and Design, Kyoto Institute of Technology, Matsugasaki Hashigami-cho, Sakyo-ku, Kyoto 606-8585 Japan

**Keywords:** Physical chemistry, Gels and hydrogels

## Abstract

We have studied an expansion behavior of pressurized bubbles at the orifice of a capillary inserted in gelator-solvent (agarose-water) mixtures as a function of the gelator concentration in which the phase transition points are included. The pressure (*P*) -dependence of the radius of the curvature (*R*) of the bubbles monitored by laser beam has shown a discontinuous decrease in the exponent (*m*) of the experimental power law *R* = *K*Δ*P*^−*m*^ (*K*: constant) from 1 to 1/2 and a discontinuous increase in the average surface tension (*γ*_ave_) obtained from the work-area plots of the mixtures exceeding that of pure water (72.6 mN/m) at 0.02 < [agarose] < 0.03 wt%, which is attributed to the disappearance of the fluidity. The apparent surface tension (*γ*_app_ = Δ*P*/2 *R*) of the system in the concentration range of 0.03–0.20 wt% has been analyzed by a modified Shuttleworth equation *γ*_app_ = *σ*_0_ + τln(*A*/*A*_0_), where *σ*_0_ is an isotropic constant component and the second term is a surface area (*A*) -dependent elastic component, in which *τ* is the coefficient and *A*_0_ is the area of the orifice. The analysis has indicated that *σ*_0_ coincides with the *γ*_app_ value of the mixture of 0.02 wt% and the second term at >0.02 wt% is the dominant component. From the appearance of the elastic component and concentration dependence of *τ*, the plateau of *τ* for the agarose-water mixtures at 0.03–0.10 wt% (Region II) has been explained to the phase separation giving two-phase mixtures of 0.02 wt% sol and 0.10 wt% gel and the upward inflection of *τ* at 0.10 wt% has been assigned to an increase in the elasticity of the gel with the increase of the agarose concentration in the range of >0.10 wt% (Region III). On considering the concentration dependence of the surface tension of agarose-water mixtures, the discontinuous and inflection points were assigned to the 1st- and 2nd-order phase transition concentrations of the agarose gel, respectively. Given the results with our tensiometry based on the optical bubble pressure method, distinct gelation points for other systems could be determined both mechanically and thermodynamically which will provide a diagnostic criterion of sol-gel transitions.

## Introduction

**Figure 1:**
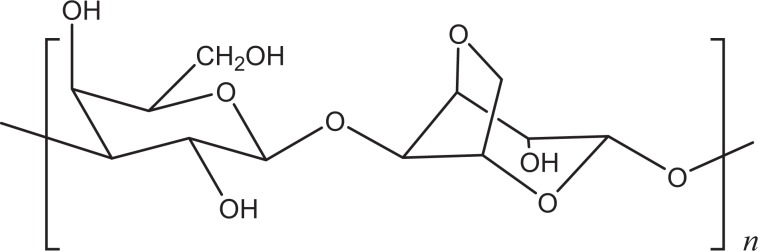
The molecular structure of agarose.

Gel is a versatile state of substances widely seen in nature, industrial products, and foods due to its solid-fluid dualism, which is owing to the high holding content of solvent in the 3D network which reveals its viscoelastic nature^1,2^. Natural and synthetic water-soluble polymers often form hydrogels where more than 90% of water is contained in weight. Polymer gels are formed by the introduction of crosslinking bonds (chemical gels) or by the mutual aggregation through an increase in the concentration or cooling of the sol (physical gel)^2^. Agarose (Fig. 1) is a polysaccharide taken from a seaweed family (Geridiaceae)^3–6^, whose molecular weight has been reported to be *M*_w_ = 0.8–1.4 × 10^5^ g/mol^7^ and is a typical substance showing physical gel formation, which has been reported to be above the concentration of 0.13 wt% at 20.0 °C^8^. However, there are several reports on the minimum gelation concentration (MGC) and sol-gel transition temperature inconsistent each other.

Sol-gel transition of physical gels has been studied intensively from mechanistic viewpoints, and several approaches by mechanical, thermal and spectroscopic measurement techniques have been conducted through the dynamic methods^1,2,9–11^. However, it remains difficult to determine the transition temperature or concentration through the dynamic rheological^8^, spectroscopic^9^, or thermal^10^ measurements owing to the variation in the definition of gel, which depends on the measurement technique employed^2^. Similarly, it is not feasible to determine MGC for physical gels as a critical phase transition concentration because of the difficulty in observing precisely the point of disappearance of fluidity and the point of the appearance of a gel state.

The mechanistic study on the gelation of polymers also has a long history of experimental and theoretical chemistry and physics^1,2,12^. Although the main mechanism of the gelation has focused on cross-linking and fibril formation, their order is not clear unless the polymer chains are not intended to be cross-linked in the preparation process of a gel. Although fibrils of linear polymers and even small molecules have been observed by transmission electron microscopy (TEM) and atomic force microscopy (AFM) techniques for flash-freeze-dried agarose gels^2^, evaporation or freezing of the solvent from the systems may induce the fibril formation, and it is not clear whether the fibril formation is essential for gelation. Although the fibril formation affords the “elasticity” or the “plasticity” to gels, which would ensure measurement of mechanical properties of the soft materials, it is not easy to assign the fibril formation as a phase transition phenomenon in a “quasi-static” thermodynamic sense. Thermal hysteresis observed during gelation also makes the analysis complicated. Although agarose gel, for example, melts at 80–90 °C upon heating, the gel is formed at 35–40 °C upon cooling^13^. Since the hysteresis could arise from the existence of several species such as aggregates with single- and double-helices and free polymer in the mixture owing to the extremely slow diffusion and relaxation processes, we planned the surface tension measurement at a constant temperature by means of the bubble pressure method, a tensiometry (surface tension measurement) used for liquids^13^, to detect the deformation of the surface of soft materials including liquids. Since the surface tension (*γ*) is defined as a ratio of a mechanical work (d*W*) needed to create a new surface area of an infinitesimal amount (d*A*), *γ* = d*W*/d*A* and this work is identical to the change in the Helmholtz energy (*F*) at constant volume and temperature or to the change in the Gibbs energy (*G*) at constant pressure (*p*) and temperature (*T*). Furthermore, since change in the surface tension is also related to the sum of the chemical potentials (*μ*_**J**_) of the chemical components (**J**) at the surface area (the Gibbs isotherm) at an equilibrium with the bulk, the mechanical measurement of the surface of a sol or a gel containing **J**’s as a solvent and a gelator can be linked directly with the thermodynamic functions of the system.

According to the Ehrenfest classification of phase transition^14,15^, continuity of 1st order derivatives of the chemical potential (*μ*) as intensive variables are supposed to be the key diagnostic criteria (see also Supporting Information). For one-component systems, *μ* is a function of *T* and *p*, *μ* = *μ*(*p*,*T*). Since *μ* requires another variable *X*_**J**_, the mole fraction of component **J** (**J** = **1**, **2**) for two-component systems, phase transition also occurs as a function of *X*_**J**_ at a finite condition of temperature and pressure. Since the surface tension is a 1st order derivative of the Gibbs energy, d*G*/d*A* for unit mole could be considered as the 1st order derivative of the chemical potential, d*μ*/d*A* which is equal to *γ* for unit mole and unit area. Therefore, some thermodynamic functions such as molar entropy can be derived from the surface tension in a quantity per unit area. For this reason, the surface tension can be a diagnostic parameter for phase transition phenomena on extending the Ehrenfest classification. For example, reported surface tension values of metals below and above the melting point are quite different each other^16^. However, the surface tension is less employed to study phase transition phenomena^17^ because of the difficulty in the measurement of the surface tension with the same tensiometry for two different phases, except for liquid-like monolayers at the liquid-air or liquid-liquid interfaces^18,19^ where the same tensiometry can be applied and changes in the surface tension can be treated with the Gibbs isotherm. The purpose of our study is to elucidate the sol-gel phase transition through a tensiometry. In other words, changes in an intensive variable *γ* will indicate the changes in other intensive variables, *i.e*. the chemical potentials upon the phase transition. However, the surface tension of gels has not been known and the Young-Laplace relationship is not assured in the gel phase. We now report the volume expansion behavior of pressurized bubbles at the orifice of a capillary inserted in gelator-solvent (agarose-water) mixtures as well as the surface tension of the mixtures as a function of the gelator concentration in which the transition point can be determined. We chose agarose as a sample of gelator because of the plentiful reference data in the literature^3–10^. In this article, the pressure-dependence of the radius of the curvature of the bubbles monitored by laser beam has been studied as a function of gelator concentration to examine the Young-Laplace relationship^20–22^ in the sol and gel states, and to establish a measurement method of the surface tension of gels. We also have studied the discontinuity and the inflection in the surface tension induced by the increase of the concentration in the mixture to examine the 1st- and 2nd-order phase transitions, respectively^14,15^.

## Results

**Figure 2:**
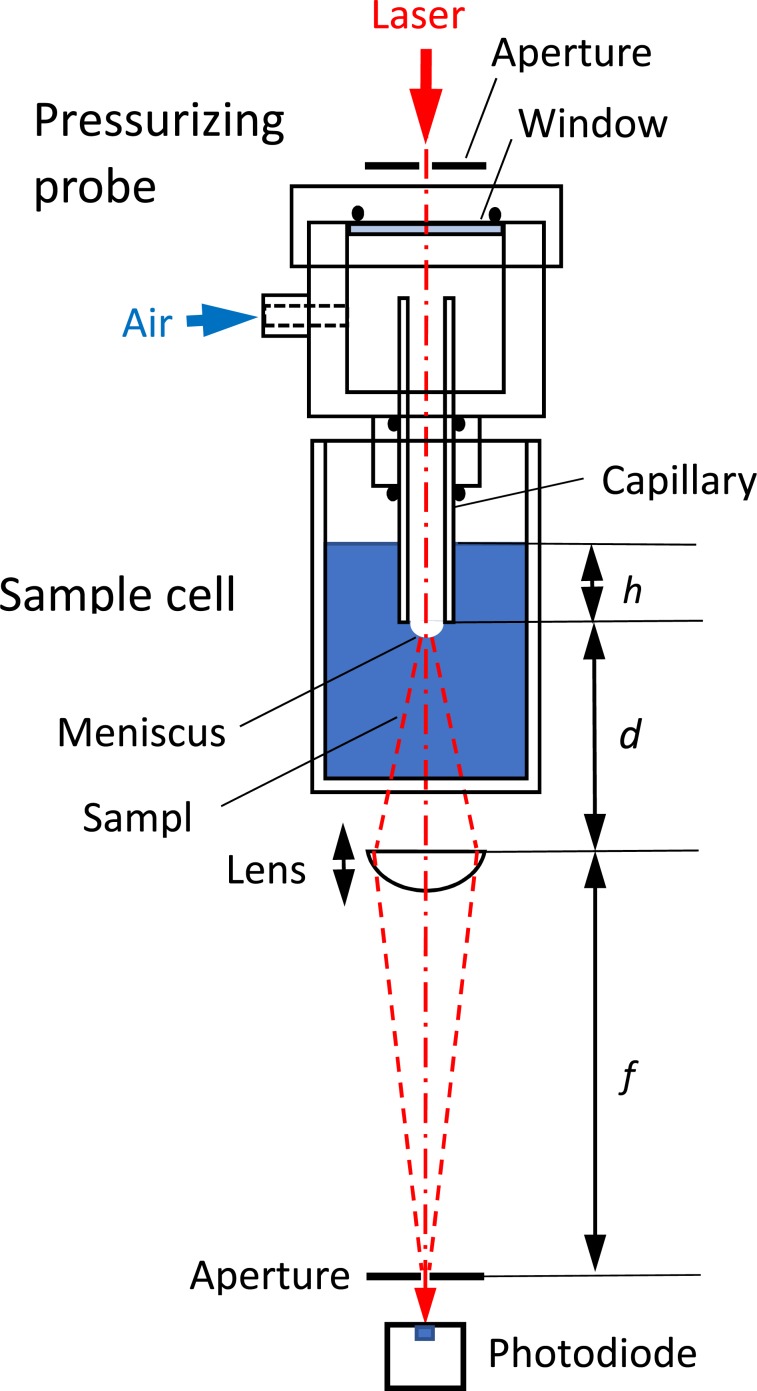
Schematic illustration of the pressurizing probe, sample cell, and other external optical items for the optical bubble pressure tensiometry, where *h*, *d*, and *f* are the distances used to calculate the radius of the curvature^30,31^. See also Supporting Information.

**Figure 3:**
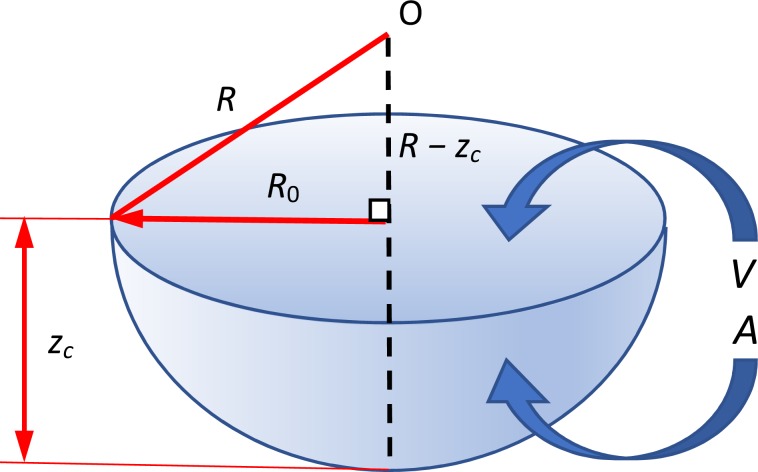
Definition of the meniscus shape with parameters *R*, *R*_0_, and *z*_c_ used to calculate *V* and *A*. See also Supporting Information (Fig. S3).

**Figure 4:**
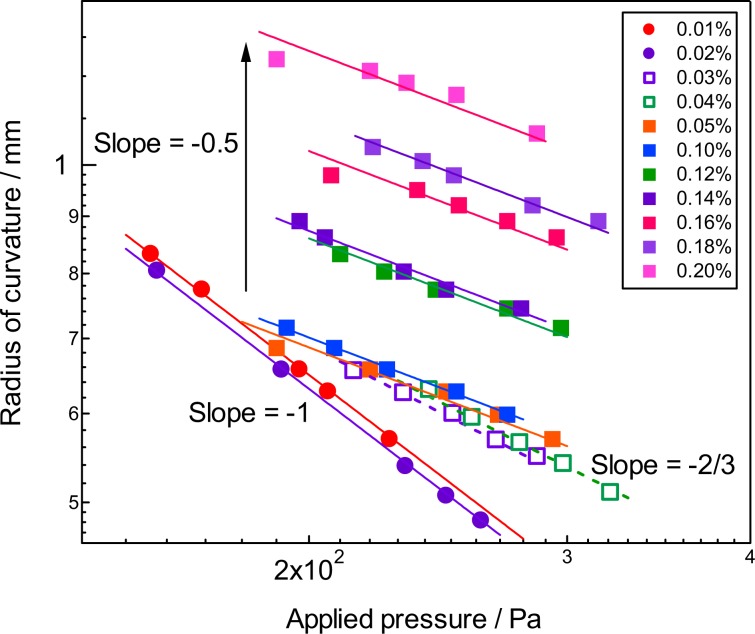
Pressure dependence of the radius of the curvature of the menisci in agarose-water mixtures at various concentrations. Figures in parentheses are the average surface tensions (*γ*_ave_).

The experimental set-up is shown in Fig. 2. The parameters which describe the shape of the bubble, radius of the curvature (*R*), surface area (*A*), volume (*V*), and depth of the meniscus (*z*_c_) are also indicated in Fig. 3. The parameters *A*, *V*, and *z*_c_ are calculated from *R* and the radius of the capillary (*R*_0_) (Supporting Information)^13^. The radius of the curvature has been measured as a function of applied pressure difference between that applied from a manometer to the bubble and the static pressure at the depth of the orifice (Δ*P*). The bubbles in the aqueous solutions of agarose with the concentrations of ≤0.02 wt% (Region I) showed a decrease of the radius upon the increase of Δ*P* obeying the Young-Laplace relationship with a constant surface tension. The surface tension was obtained by a nonlinear least square curve fitting showing a gradual decrease from that of pure water (72.6 mN/m) to 63.4 mN/m at 0.02 wt% with the agarose concentration as observed for amphiphilic polymers such as polyethylene oxide. The decrease of the radius of the meniscus for the mixtures of ≥0.03 wt% by the applied pressure became smaller compared to that for the dilute solutions, although the appearance of the mixture was solution-like. The double logarithmic plot of *R* versus Δ*P* indicated a slope of −1 for the solutions and a slope of −1/2 for the mixture of ≥0.05 wt%, and mixtures of 0.03 and 0.04 wt% showed an intermediate value of the slope of ≈−2/3. This abrupt change in the *R*—Δ*P* power law behavior of the bubble strongly suggests that the bubble surface of the mixtures of ≥0.03 wt% is no longer liquid-like (Fig. 4).

**Figure 5:**
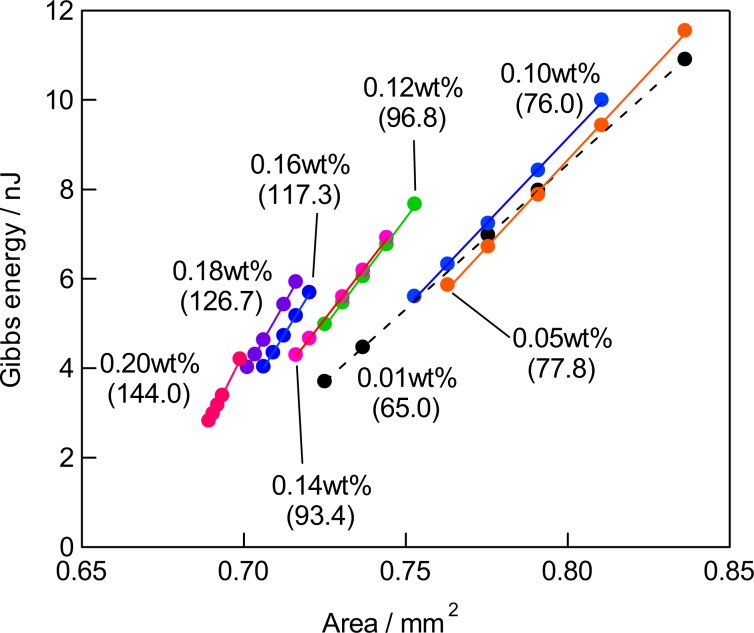
The Gibbs energy change as a function of the surface area of the meniscus corresponding to Fig. 4.

**Figure 6:**
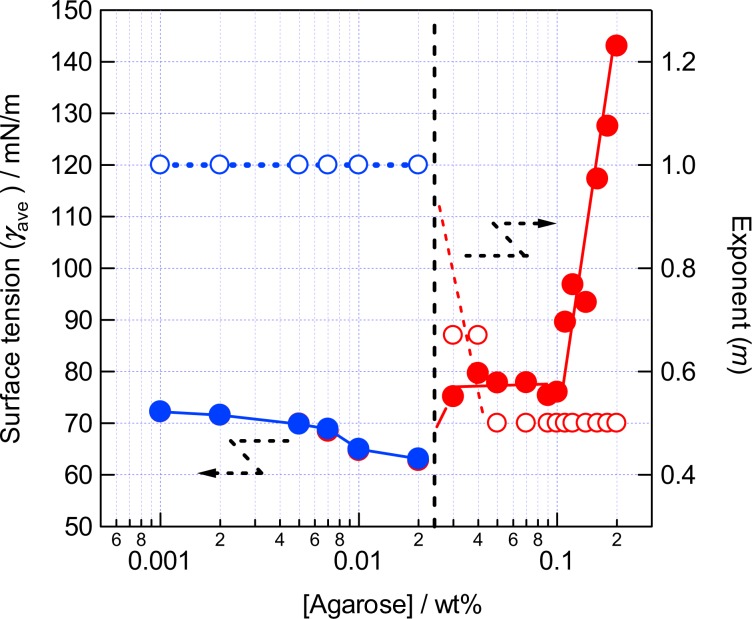
The average surface tension (*γ*_ave_) of agarose-water mixtures at various concentrations (left axis) and the exponent (*m*) of the power law of the radius of the curvature (*R*) *R* = *K*(Δ*P*)^−*m*^ corresponding to the slope in Fig. 4 (right axis) as functions of the agarose concentration.

To compare the surface property of the mixture of ≥0.03 wt% with that of solutions of ≤0.02 wt%, we estimated the surface tension of the mixture without using the Young-Laplace relationship throughout the *R*—Δ*P* sets at the agarose concentrations. Assuming the spherical surface of the meniscus, the radius of the curvature (*R*) can been converted to the surface area (*A*) and the volume (*V*) of the meniscus, and the *R*—Δ*P* curve has been translated into a Δ*P*—*V* curve, then accumulation of the Δ*P*—*V* curve gives a course of to the Gibbs energy change (Δ*G*) as the meniscus has done the work (Δ*W*) for the volume expansion against the surface tension of the agarose-water mixture. The plot of Δ*G* vs *A* shows straight lines for all the mixtures (Fig. 5). Then, the slopes of the lines indicates the average surface tension of the system (*γ*_ave_) as defined thermodynamically, *γ* = (∂*G*/∂*A*)_*T,p*_. The error in the surface tension of the solutions with that obtained by the nonlinear least square method is less than 1% (±0.1 mN/m). The surface tension estimated from the Gibbs energy change has a slightly larger error (±0.2 mN/m) than that obtained by the non-linear curve fitting due to the trapezium approximation of 5 sets of the data points in the integration of the Δ*P*—*V* curve. Nevertheless, the surface tension values obtained by the two calculations for the solution with concentrations of ≤0.02 wt% agreed within 0.2%. The average surface tension plotted vs. agarose concentration clearly indicates a jump from 63.4 to 75.0 mN/m between 0.02 and 0.03 wt% (Fig. 6). The surface tension of the 0.03 wt% mixture exceeds that of pure water (72.6 mN/m measured with our system) ruling out the exuding of pure water from the 3D network. The surface tension of the mixture is almost constant up to 0.10 wt%. However, it increases for the mixtures of >0.10 wt% until 154.7 mN/m for the 0.20 wt% mixture, but our measurement system was not able to measure the surface tension of mixtures of >0.20 wt%. These results clearly indicate that the mixtures undergo the sol-gel phase transition and the expansion behavior of the bubbles in the gels is network-limited.

## Discussion

**Figure 7:**
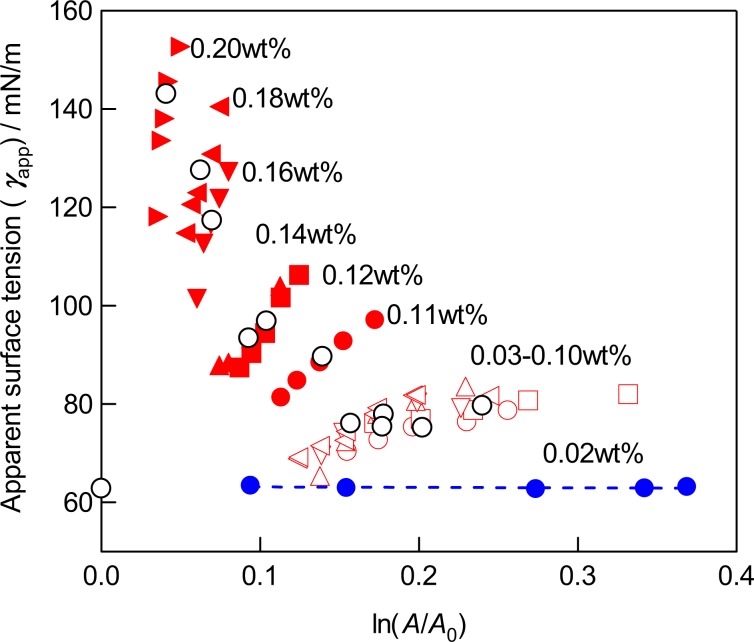
A plot of *γ*_app_ vs. ln(*A*/*A*_0_). Note that blue filled circles for the mixture of 0.02 wt% indicate the apparent surface tension is independent of the surface area *A*. Black open circles indicate the *γ*_ave_ values obtained by the *G*—*A* plots and are plotted against the average surface areas of the menisci (*Ᾱ*). For the mixture of 0.02 wt%, the *γ*_ave_ value is plotted against *A* = *A*_0_ to indicate *γ*_ave_ = *σ*_0_ (see text).

As the Young-Laplace relationship has been derived from the equation concerning the work for the volume expansion and that for the increase of the surface area of a bubble with a small change in the radius (d*R*), it is necessary to consider the work for the volume expansion for the agarose-water mixtures of ≥0.03 wt%. On considering the gel as a solid, we cannot apply the equation with a constant surface tension, d*W* = d*Aγ* because the presence of the surface strain due to the elasticity. However, the Young-Laplace equation must hold at a given Δ*P*. We have calculated the surface tension for each *R*—Δ*P* data set as apparent surface tension (*γ*_app_) and reconsider the surface tension with the equation d*W* = d(*A*σ) = σd*A* + *A*dσ, where σ is the thermodynamic surface tension. Differentiation of this equation with respect to *A* gives an equation *γ*_mec_ = σ + dσ/dln*A* according to the treatment of the surface tension of solids^16,17^. As shown in Fig. 7, *γ*_mec_ increases linearly with the increase of ln(*A*/*A*_0_) and tends to converge to the *γ*_mec_ value for the 0.02 wt% solution (*γ*_mec_ = 63.4 mN/m) upon extrapolation of *γ*_mec_ values for various mixtures at zero expansion (*A* = *A*_0_). The intercepts are almost independent of the agarose concentration, while the slope is dependent on the agarose concentration. Therefore, we can rewrite the equation for *γ*_mec_ as *γ*_mec_ = σ_0_ + dσ/dln(*A*/*A*_0_), where σ_0_ = 63.4 mN/m. The average surface tension *γ*_ave_ can be considered as a representative value of *γ*_mec_ at the average surface area (*A* = *Ᾱ*), which is dependent on the applied pressure within the fracture limit of the bubble at an agarose concentration.

We now return to the *R*—Δ*P* relationship. The experimental power law observed for the radius of the curvature to the applied pressure showed an exponent of −1/2, *i.e*. *R* = *K*(Δ*P*)^−1/2^, where *K* is the constant. This relationship could be obtained from the Young-Laplace equation *R* = Δ*P*/2*γ*_mec_ and the equation for *γ*_mec_ at the corresponding surface area *A* = *π*(*R*_0_^2^ + *z*_c_^2^). Although we did not obtain the analytical solution for *R* = *R*(Δ*P*), the second term (compressibility modulus) in *γ*_mec_ originating from the surface strain appears and dominates with the increase of the agarose concentration above the minimum gel concentration and this will cause the change in the exponent. We also obtained a linear relationship between *K* and *γ*_mec_ in their double logarithmic plot (Fig. S5). As a result of the introduction of the surface strain term for the analysis of the *R*—Δ*P* data, we could conclude that the observed change in the exponent *m* is phenomenal although detailed numerical analysis might reveal the *R*—Δ*P* relationship.

**Figure 8:**
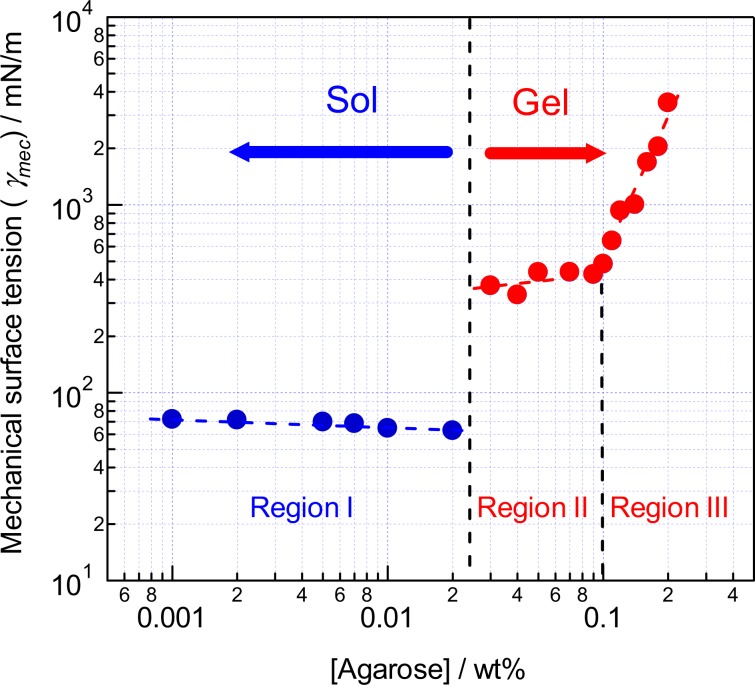
A plot of *γ*_mec_ vs. agarose concentration, which is divided into 3 regions (Region I, II, and III. See text) owing to the behavior of *γ*_mec_.

We have introduced the surface strain term dσ/dln(*A*/*A*_0_) in the mechanical surface tension of agarose-water mixture with the concentration of >0.02 wt% and now show a plot of *γ*_mec_—concentration (Fig. 8). The introduction of the surface strain term indicates the abrupt increase of *γ*_mec_ at 0.02 < [agarose] < 0.03 wt% and an inflection at 0.10 wt% more clearly as compared to the *γ*_ave_—concentration plot (Fig. 6). As shown in Fig. 8 (and Fig. S6), a plateau is seen in the concentration range of 0.03–0.10 wt% (Region I). According to Herring, *γ*_mec_ contains a scalar and a tensoric terms, the latter is corresponding to the surface strain term. Rusanov^23,24^ explained nonequivalence of the mechanical (*γ*_mec_) and thermo-dynamic (*σ*) surface tension for wetting of an isotropic solid surface co-existing mobile components by relating *σ* to *γ*_mec_ and the sum of the chemical potentials of the mobile (**I**) and immobile (**J**) components d*σ* = − *s*_(**J**)_d*T* + (*γ*_mec_ − *σ*)dln*A* − Σ*Γ*_**I**(**J**)_*μ*_**I**_, where *s*_(**J**)_ is the entropy surface density and *Γ*_**I**(**J**)_ is the surface excess of the mobile component at the surface of the immobile component. For the present case, **I** = water and **J** = agarose, Σ*Γ*_**I**(**J**)_*μ*_**I**_ can be considered to be independent of *A* and the experiment has been conducted at the constant temperature. Therefore, we obtain *γ*_mec_ = σ + dσ/dln*A* = σ_0_ + dσ/dln(*A*/*A*_0_) again on considering *γ*_mec_ = σ_0_ at *A* = *A*_0_. This means the mechanical surface tension is consisted of scalar term as the surface tension of *σ*_0_ value for the 0.02 wt% solution and the strain term dσ/dln*A* due to the strain of the agarose gel as a solid.

The strain term is also related to the chemical potentials of the immobile components. However, there is no fast diffusion and no equilibrium between the surface and the bulk phase meaning the inequality of the chemical potentials and there is an area-dependent strain due to the measurement. For this reason, we cannot treat the strain term directly as a parameter for the phase behavior of the solid. Fortunately, the elasticity of polymer gels originates from the entropy of the polymer chain, i.e. Δ*F* = −*T*Δ*S*^25^. Now we can treat the strain term as the entropy surface density of the immobile component, *i.e*. the gel network. With a small isotropic deformation of the surface area, experimental value of the strain term is given by (dσ/dln*A*)ln(*A*/*A*_0_) ≈ 2*ετ*, where *ε* is the isotropic tensor and *τ* = dσ/dln*A*. Therefore, the mechanical surface tension *γ*_mec_ can be a thermodynamic quantity of gels and inflection of the strain term is attributable to a change in the elasticity due to a structural change of the gel. We have concluded that the surface tension can be a criterion for sol-gel transition: the observed discontinuity explained by the appearance of the strain term is assigned to the sol-gel phase transition as the 1st-order phase transition is, and the inflection of *τ* reflects the change in the elasticity of the solid gel phase as the 2nd-order phase transition.

Finally, we will enter the phase behavior of the agarose-water mixture. The surface tension of the mixture of ≤0.02 wt% (Region I) is the liquid with no doubt because of the Young-Laplace behavior and *γ*_mec_ = *σ* throughout the applied Δ*P* range. The observation of the plateau in *γ*_mec_ in the range of 0.03–0.10 wt% (Region II) indicates that the strain term of the gel is almost constant. This strongly suggests that the chemical composition of the gel is almost identical despite of the change in the net concentration. As judged from the above discussion on *σ*_0_, the chemical composition of the sol is also constant. Therefore, only quantities of both phases seem to vary with the concentration. This has invoked the idea of phase separation and the lever rule to explain the slight increase of *γ*_mec_ in Region II. Spinodal decomposition of the mixture followed by gelation and concomitant phase diagrams have been reported for the mixture of atactic polystyrene in cyclohexane as judged by the test tube tilting and ball-drop method^26^. Indei has reported network formation of poly(vinyl alcohol)-borax by percolation as judged by the micro-rheology^27^. Similar mechanisms for gelation of the agarose-water system at lower concentrations have been proposed on the basis of the rheological^8^ and dynamic light scattering measurements^28^. Spinodal decomposition of agarose-water at 0.02 < [agarose] < 0.03 wt% gives 0.02 wt% solution and 0.10 wt% gel whose amounts are determined by the lever rule as an origin of *σ*_0_ and weak concentration-dependence of *γ*_mec_ in Region II. This leads us to a conclusion that the gel in Region II is two-phase gel with a percolated or a fractal-like structure because of the freedom of the system for intensive variables (*f*) is 0 for the sake of the phase rule (*f* = *c* – *p* + 2, where *c* and *p* are the numbers of components and phases, respectively. Note that two of *f* are occupied by the temperature and pressure of the experimental condition.). The slight increase in *γ*_mec_ can be explained by the number of the cross-linking points which increases with the increase of the amount of the gel domain in the mixture.

**Figure 9:**
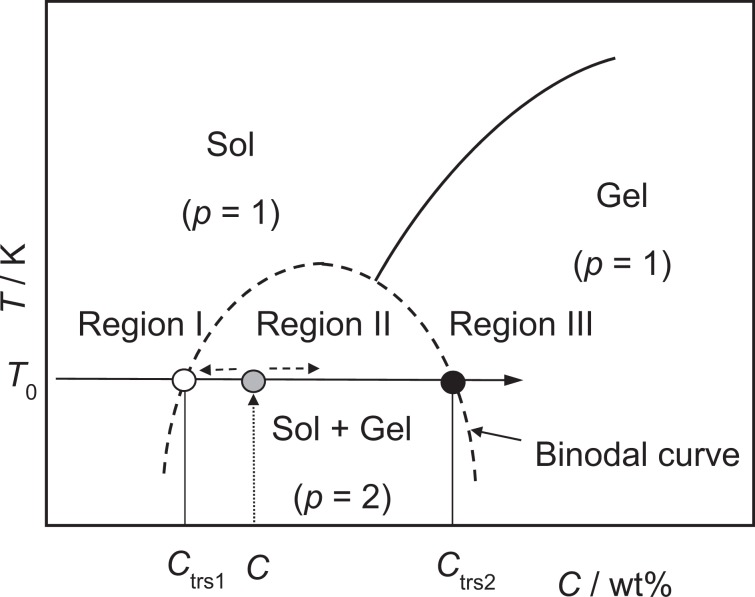
A schematic phase diagram that explains the observed surface tension behavior^27,28^. The critical concentrations *C*_trs1_ and *C*_trs2_ are the crossing points of *T* = *T*_0_ (20 °C) and the bimodal curve. The amounts of agarose in the sol and gel phases (with concentrations of *C*_trs1_ and *C*_trs2_, respectively) formed upon the phase separation of a mixture at a given concentration of *C*_0_ in Region II are determined to be in a ratio of *C*_trs2_ − *C*_0_: *C*_0_ − *C*_trs1_ owing to the lever rule.

As Rees predicted that agarose molecules form double helix aggregates^5^, which have been observed by X-ray diffraction and other micro-imaging techniques with ≥0.1 wt% mixtures, Liu *et al*. proposed a “primary fiber” as an intermediate in the gelation of agarose^6^. The primary fiber could be an aggregate formed by twisting of two agarose chains which act as a substrate for self-epitaxial nucleation to form the 3D network. Our observation with the mixtures in Region II and the >0.10 wt% concentration region (Region III) strongly suggests that the expansion of the network requires some energy to unwind the primary double helix, *i.e*. the energy to break the hydrogen and the hydrophobic bindings and the energy to unwind the multiple helix requires increases by the increase of the concentration. Tieleman *et al*. have measured the area-dependence of the surface tension of a lipid monolayer, which undergoes phase transition by area expansion^29^. They have reported the compressibility moduli (corresponding to *τ* = dσ/dln*A*) of dipalmitoyl-phosphatidylcholine (DPPC) to be 1400 ± 20 mN/m for liquid condensed phase at a molecular area (*A*_L_) of *A*_L_ = 0.475 nm^2^, 200 ± 12 mN/m for liquid condensed and liquid expanded coexisting phase at *A*_L_ = 0.58 nm^2^, and 100 ± 20 mN/m for liquid expanded phase at *A*_L_ = 0.620 nm^2^, respectively^29^. They have also observed inflection points in the *γ*-*A* plot^29^. On the other hand, our observation indicated the values of dσ/dln*A* = 300–500 mN/m in Region II and 500–3500 mN/m in Region III together with the inflection point at 0.10 wt% (Fig. S6). These data clearly show that the two-phase gel in Region II has a weak binding nature as compared to the monolayer of alkyl chains. Upon increasing the agarose concentration, dσ/dln*A* values of the gel increase drastically by the increase of the density of cross-linking points^12^. This means that the structure of the gel changes with the concentration and the freedom *f* = 1 corresponding one-phase gel (*p* = 1) in Region III. Although melting of the agarose gels of >0.10 wt% (Region III) is well known to occur at 35–50 °C^5,6^, the systems below this concentration (Region II) have been reported to be a suspension of micro-gel^8,28^, and its phase transition behavior upon the concentration change has been mentioned less frequently. Although intensive study including temperature dependence is needed to draw an exact phase diagram, we show a schematic phase diagram which explain the observed behavior of the surface tension as a function of agarose concentration (Fig. 9).

## Material

Agarose (Agarose-S tablets, Nippon Gene, for electrophoresis use, sulfate (−SO_4_) content ≤0.1%) was used as received. Its molecular weight of Agarose-S as given by the manufacturer was 2–3 × 10^5^ g/mol determined by liquid chromatography. Mother solution of agarose (1.0 wt%) was prepared by heating a mixture of an agarose tablet (≈0.50 g) and distilled deionized water (500 mL) was heated to 90–95 °C in a beaker with a microwave oven. The solution was diluted at ≈40 °C (above the gelation point of 37–39 °C for 1.5 wt% solution).

## Method

We have modified the bubble pressure method where the experimental apparatus possesses a Teflon cell holding a capillary and an optical window which enable the pressurization of the sample and monitoring of the radius of the curvature (*R*) of the meniscus (bubble) optically with a laser beam passing through the capillary (pressurizing optical probe)^30,31^. The meniscus formed in the solution or gel acts as a concave lens for the laser beam. The focal length of the meniscus is determined by an external optics and a photodetector system. The radius of the curvature was translated into the surface tension of the mixture, as a first derivative of Gibbs energy (*G*) with respect to the surface area (*A*), through the translation into the surface area and volume (*V*) of the meniscus as a function of the pressure applied (*P*). The gelation has been judged by the apparent surface tension exceeding that of pure water. The details are described in the Supporting Information.

## Conclusion

In conclusion, we have established a tensiometry for weak gels using the optical bubble pressure method through the demonstration of the sol-gel transition behavior of the agarose-water mixtures as consecutive occurrences of the loss of the fluidity and the increase of the surface tension upon the increase of the gelator concentration, which can be attributed to the 1st- and 2nd-order phase transitions through the analyses of the Gibbs energy change of the meniscus for its expansion and the mechanical surface tension for solids as introduced by Shuttleworth^32^ and Herring^33^, and with the aid of the concept of entropic elasticity. Therefore, the increase in the surface tension upon the gelation can be attributed to the changes in the mechanical properties of the polymer network as a solid with entropic elasticity and can be interpreted into thermodynamic phase behaviors. The present results has indicated that the surface tension measurement will provide a reliable criterion for the sol-gel transition of other polymeric systems and also for that of low molecular-mass organic gelator systems, which are being increasingly studied^34,35^.

## Supplementary information

Supplementary information
