# Supplementary material for: Concentration dependence of the sol-gel phase behavior of agarose-water system observed by the optical bubble pressure tensiometry
Source: Sci Rep. 2020 Feb 14;10:2620. doi: 10.1038/s41598-020-58905-8 (PMC7021719; doi:10.1038/s41598-020-58905-8)
Supplement: Supplementary file 1 — Supplementary information [file 41598_2020_58905_MOESM1_ESM.pdf]

**Supplemental information**

# Concentration dependence of the sol-gel phase behavior of agarose-water system observed by the optical bubble pressure tensiometry

Nobuyuki Ichinose,* and Hodaka Ura

## Experimental

### Set-up of the tensiometer

The set-up for our tensiometry is described in ref 32 and 33 except for some modification (Figure S1). A glass capillary with a 30 mm length and an inner diameter of 0.92 mm (Hirschman, Minicaps, 20 μL) was fixed with two O-rings to a Teflon holder. The holder was placed to a gas-tight Teflon cell holding a quartz window (30 mm diameter, 1 mm thickness). The capillary was immersed in the liquid filled in a cylindrical quartz optical cell (30 mm diameter, 30 mm height, 2 mm thickness window at the bottom) to the depth of $h$ = 10.0 mm by lifting a sample cell with a rack-and-pinion stage of a precision of 0.1 mm. The static pressure ($P_s$) operating to the meniscus is given by the depth $h$ as $P_s = P_{out} = \rho gh$, where $\rho$ is the density of the sample liquid. A gas inlet was connected to a manometer using silicone tubing equipped with a T-joint and a syringe to pressurize the capillary. An aqueous solution of sodium dodecyl sulfate (SDS, [SDS] = 5 × 10 mol dm$^{-3}$, density: $\rho_0$ = 0.99889 g cm$^{-3}$ at 20 °C) was used as a gauge liquid of the manometer. The pressure applied to the meniscus ($P_{in}$) was measured by the manometer with a precision of 1 Pa. A laser beam from a semiconductor laser (650 nm, 0.2 mW, ≈0.9 mm diameter) was passed through an aperture (A1, ≈0.8 mm diameter) and the center of the capillary. The dispersed beam was focused by an external plano-convex lens (L, focal length of $f_2$ = 100.0 mm, diameter of 50 mm) onto another aperture (A2) in the back where a silicon photodiode (Hamamatsu, S6675, bias voltage: 3.0 V) as a detector was placed as to maximize output voltage when the beam is focused onto the aperture. The diameter of the laser beam was also adjusted by means of an iris (I) in a range of 0.1-0.8 mm. The use of the whole meniscus as a lens was avoided to remove the spherical aberration caused by the peripheral part which may contain the effect of the interaction between the capillary wall and the sample, and the central part was used by adjusting the iris to select the diameter of the laser beam of ≈0.7 mm. The beam profile of the laser was not Gaussian and used without any modification by optical devices such as telescope. The output of the photodetector was monitored by a digital oscilloscope (Iwatsu DS-5320) at a rate of 10 Hz. When a gas pressure was applied to the capillary, a bubble grew and departed from the orifice. The gas pressure was gradually increased and noted, then, the position of the external lens was adjusted with a precision of 0.1 mm to maximize the output of the photodetector. The back focal length (BFL, $f$) of the combination of the meniscus and the lens can be determined from the positions of the meniscus and the external lens from the aperture (eq S1), where $d$ is the distance between the meniscus and the lens L.

$$\mathrm{BFL} = f_2(d - f_1)/\{d - (f_1 + f_2)\} \qquad \text{(S1)}$$

From the negative focal length $f_1$, the radius of the meniscus ($R$) can be derived using a value of refractive index of the liquid ($n$) by eq S2.

$$R = -(n - 1)f_1 \qquad \text{(S2)}$$

The measured focal length was corrected with the thickness of the window of the optical cell (quartz, $d'$ = 2 mm, $n_D^{20} = n'$= 1.458). The insertion of the window into the optical path shifts the imaginary focal point of the meniscus (eq S3).

$$f_1 = \{\mathrm{BFL}(d - d'/n') - f_2\{\mathrm{BFL} + d - d'/n')\}/(\mathrm{BFL} - f_2) \qquad \text{(S3)}$$

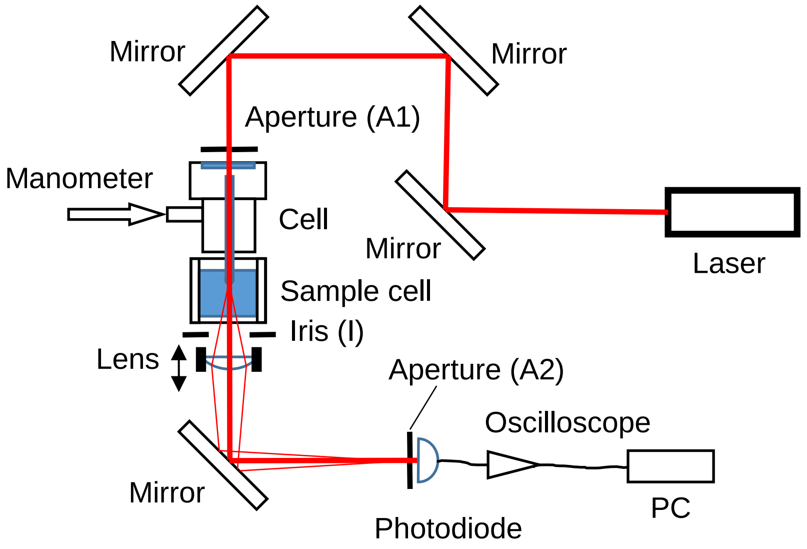


**Figure S1.** The set-up of the tensiometer using a pressurizing capillary and a laser for monitoring the radius of the curvature of the meniscus. The diverged laser beam by the meniscus is focused onto an aperture (A2) in front of the photodiode. Holders and posts of the optical components are omitted.

**Measurement of surface tension of water and agarose-water mixtures**

We considered the refractive indices of the samples and glasses used in this study for 650 nm light to be the same as those for 589 nm light ($n_D^{20}$'s). Refractive indices of the samples were measured with a refractometer (Atago, PAL-R1). Densities of the samples (ρ) were measured for 20 mL of the samples by weighing at 20 °C. The temperature of the sample was the same as the room temperature which was controlled by a usual air-conditioner within ±0.5 °C. The sample was left at the room temperature for at least 15 min prior to the measurement. The sample cell was placed in the optical chamber and also covered with aluminum foil to prevent the air stream on the sample surface, which blows the vapor of the sample causing the decrease of the surface temperature and evaporation or invasion of the moisture in the sample or in the air. The air pressure was applied to the meniscus through the silicone tube from a 1-mL syringe, which was replaced with another one with a capacity

of 5 mL or 0.5 mL depending on the surface tension of the samples. Surface tension ($\gamma$) of the liquid sample was determined by the curve fitting of 5 pressure difference ($\Delta P = P_{in} - P_{out}$) — radius ($R$) points with the Young-Laplace equation ($R = 2\gamma/\Delta P$). However, this could not be applied to the surface tension of the gel samples because the obtained $R$— $\Delta P$ curve did not obey the Young-Laplace equation throughout the applied $\Delta P$ range (see text) (Figure S2).

The observed change in the radius of the curvature of the meniscus $R$ can be considered to be a result of the change in the depth of the meniscus $z_c$. Since the meniscus is a part of the sphere with a radius of $R$ and its cross section has a radius of the capillary ($R_0$), the depth $z_c$ can be estimated using eq S4 (Figure S3).

$$z_c = R - (R^2 - R_0^2)^{1/2} \qquad \text{(S4)}$$

Surface area ($A$) and volume ($V$) for a meniscus are given by eq S5 and S6 as functions of $z_c$ and $R_0$.[19,32]

$$A = \pi(R_0^2 + z_c^2) \qquad \text{(S5)}$$

$$V = \pi(3R_0^2 z_c + z_c^3)/6 \qquad \text{(S6)}$$

The Gibbs energy change ($G$) for the increase of the volume of the meniscus ($V$) relative to the plane of the orifice upon the increase of the applied pressure difference ($\Delta P$) and was calculated by the point-to-point calculation of the area of the $\Delta P$—$V$ curve (eqs S7 and S8)

$$G(1) = \Delta P(1)V(1)/2 \qquad \text{(S7)}$$

$$G(i) = G(i-1) + \{\Delta P(i) + \Delta P(i-1)\}\{V(\mathrm{i}) - V(i-1)\}/2 \quad (i = 2\text{-}5) \quad \text{(S8)}$$

, where $i$ is the number of the point. Data analysis was performed by means of a PC software (Igor Pro ver. 6, Wave Metrics). The obtained Gibbs energy of the meniscus was plotted versus the surface area (Figure S4), which was corresponding to the $R$— $\Delta P$ curve as shown in Figures S2. The Gibbs energy was plotted versus the surface area calculated with eq S5 (Figure S5). The plot showed a linear relationship whose slope corresponds to the average surface tension of the gel ($\gamma_{ave}$) due to the relationship in eq S9, the definition of the surface tension.

$$\gamma = (\partial G/\partial A)_{T,p} \qquad \text{(S9)}$$

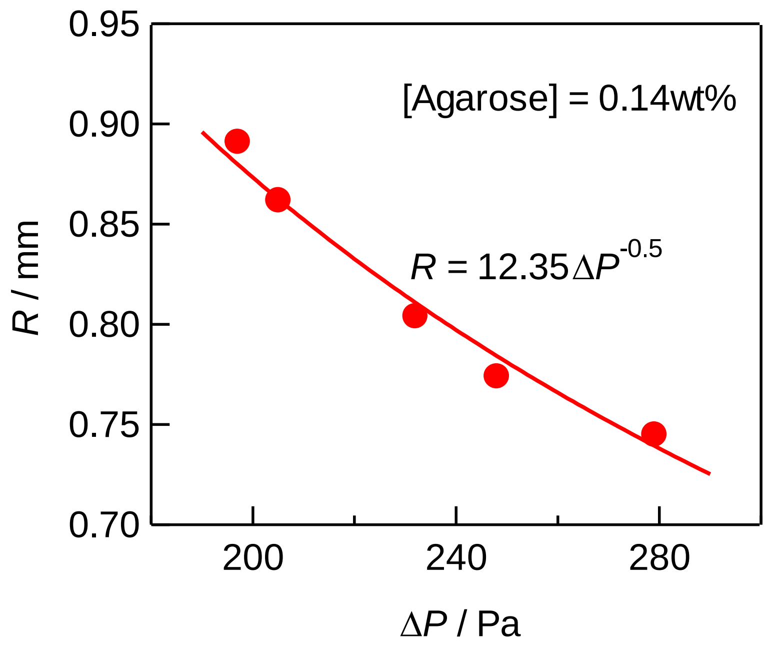


**Figure S2.** The observed radius of the curvature of the meniscus ($R$) versus applied pressure difference ($\Delta P$) for a mixture of agarose-water (agarose content 0.14 wt%) at 20°C. The solid line is the best-fit curve for the power law $R = K \cdot (\Delta P)^{-m}$, ($m$ = 0.5) (see text).

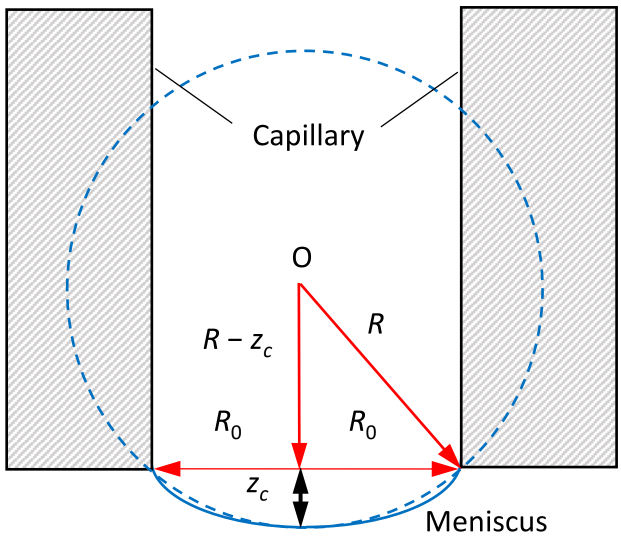


**Figure S3.** A schematic illustration of the meniscus which is approximated as a part of a sphere.[32] O and $R$ are the centre and the radius of the sphere, respectively. $R_0$ is the radius of the capillary. The depth of the meniscus $z_c$ is defined as a parameter to calculate the volume $V$ and surface area $A$ of the meniscus.

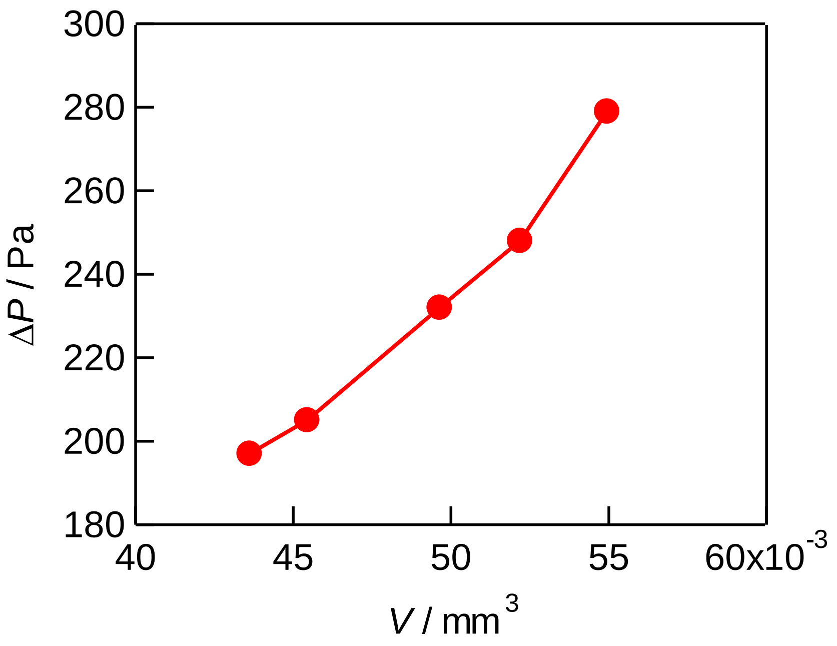


**Figure S4.** A plot of the applied pressure difference (Δ*P*) versus volume of the meniscus (*V*) calculated from the radius of the curvature of the meniscus with eq S6 for a mixture of agarose-water (agarose content 0.14 wt%) at 20°C.

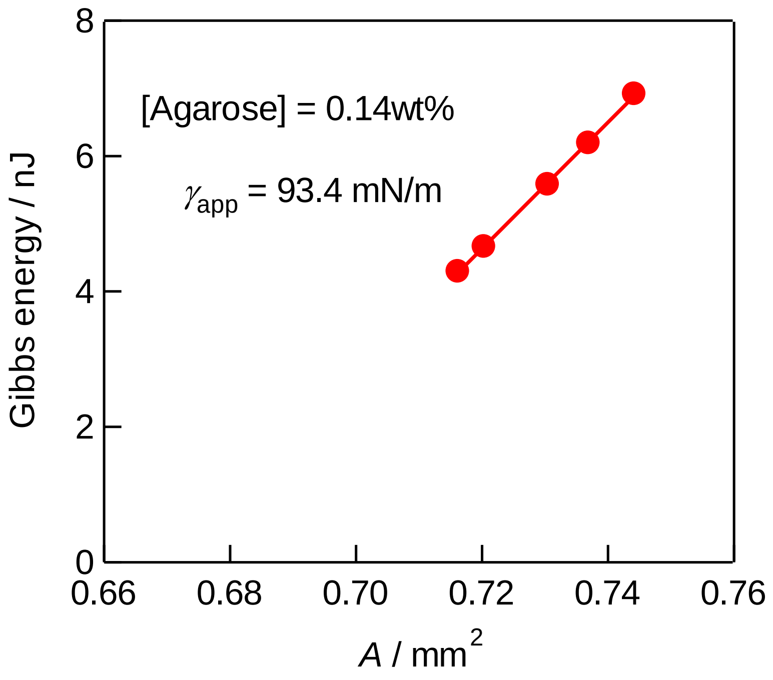


**Figure S5.** A plot of the Gibbs energy of the meniscus (*G*) versus surface area of the meniscus (*A*) calculated from the radius of the curvature of the meniscus with eq S5 for a mixture of agarose-water (agarose content 0.14 wt%) at 20°C.

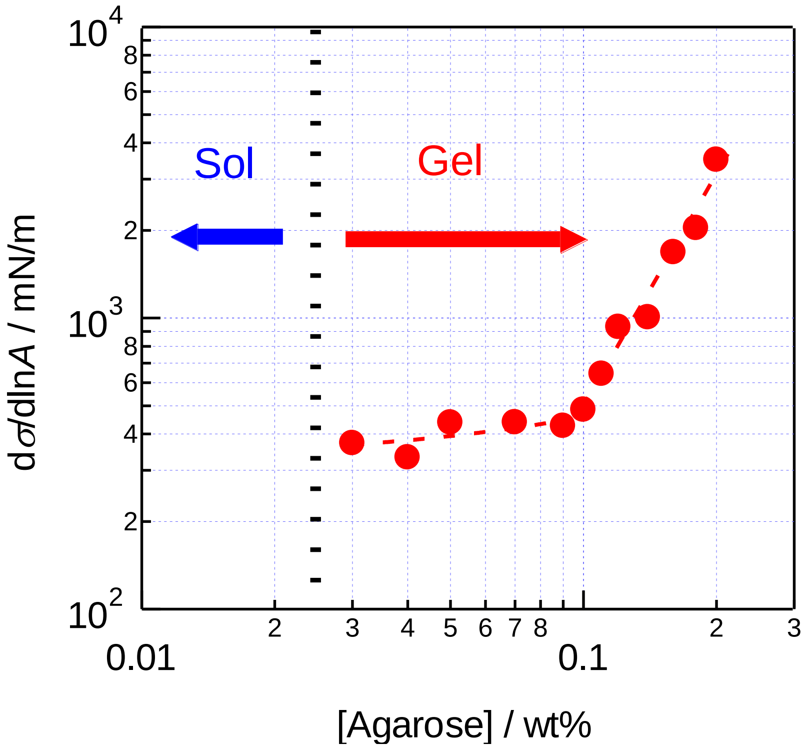


**Figure S6.** A plot of the surface strain term d$\sigma$/dln$A$ vs. agarose concentration.

**Discontinuity of the surface tension at the phase boundary**

As shown in Figure 5, discontinuity and inflection of the surface tension of agarose-water mixture were observed. We have to mention for the use of the terms, 1st- and 2nd-order transitions for the binary system and for the assignment of the discontinuity and inflection with respect to the concentration of agarose to the 1st- and 2nd-order transitions, although the original Ehrenfest's classification of phase transition deals with single component systems based on the first and second derivatives of the Gibbs energy with respect to temperature or pressure. A λ-transition in the heat capacity of Cu-Zn alloy (β-brass) is exemplified for the 2nd-order phase transition of a binary mixture in a recent textbook of physical chemistry (ref. S1). We also mention for the parameter to describe the phase transition is the chemical potential ($\mu$) due to its intensive nature and not the Gibbs energy in the textbook (ref. S1).

The observed discontinuity of the surface tension at the concentration ≈0.025 wt% does not assure mathematically the discontinuity of the surface tension at 20°C with respect to the temperature. However, it is a cross point of the binodal curve with the $T$ = 20°C line and this point obviously indicates the transition concentration. Since the surface tension $\gamma$ has a negative gradient toward temperature $\gamma$(a') < $\gamma$(a) in general and is discontinuous at the point ($T$ = 20°C and $c$ = ≈0.025 wt%) $\gamma$(a) < $\gamma$(b), then, we will obtain similar discontinuity of $\gamma$(b) < $\gamma$(b') at $T$ = 20°C at the limit of $T_0$ =

20−$\delta$°C and $T_1$ = 20 + $\delta$°C ($\delta \approx 0$) and $C_0 = C_1 - \varepsilon$ ($\varepsilon \approx 0$) although $\gamma$(a) ≈ $\gamma$(a’) because two paths of (a→b) and (a→a’→ b’→b) are isoergonic processes and require the latent heat (Figure S7).

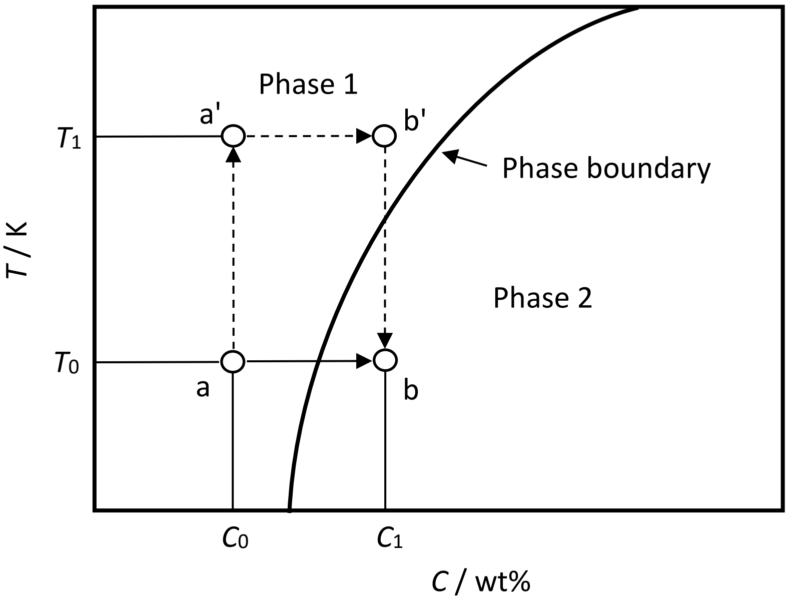


**Figure S7.** A schematic temperature ($T$) -concentration ($C$) phase diagram for agarose-water mixture. Phase 1 and Phase 2 exhibits different concentration dependence of the surface tension. A concentration change process (a→b) can be replaced with a 3-step process (a→a’→ b’→b) because of the path-independent nature of thermodynamic state functions.

## References


S1. P. Atkins, J. de Paula, *Atkins’ Physical Chemistry*, 10th Ed., Oxford (Oxford, UK), 2014, Chapter 4.
